# Supplementary material for: Lipopolysaccharide-affinity copolymer senses the rapid motility of swarmer bacteria to trigger antimicrobial drug release
Source: Nat Commun. 2018 Oct 15;9:4277. doi: 10.1038/s41467-018-06729-6 (PMC6189052; doi:10.1038/s41467-018-06729-6)
Supplement: Supplementary file 1 — Supplementary Information [file 41467_2018_6729_MOESM1_ESM.pdf]

Supplementary Information

Lipopolysaccharide-affinity copolymer senses the rapid motility of swarmer bacteria to trigger antimicrobial drug release

Lu et al.

Supplementary Notes

Supplementary Note 1: The lower critical solution temperature (LCST) of copolymer **3** was determined by measuring the transmission of the copolymer in PBS buffer (pH = 7.2). Supplementary Fig. 2 shows separately the transmission of copolymer **3** and pNIPAAm (α,ω-bis(carboxy)-terminated) at 450 nm. The transmission (*T*) of completely solubilized copolymer **3** (*T* ~ 94) is slightly lower than that of solubilized pNIPAAm (*T* ~ 100) due to small absorbance of light by the peptide ligand in the former. By defining the LCST (cloud point) of the polymer to be the temperature at which the transmission (at 450 nm) decreases to 50% of the initial transmission, the LCST of pNIPAAm and copolymer **3** are determined to be 30 and 53 ^oC, respectively. It is pertinent to note that the LCST of copolymer **3** is significantly higher than the temperature used in the MIMS experiments (≤ 37 ^oC), and the copolymer does not precipitate out at the temperature range from 25 to 46 ^oC. Similar observation was also seen when the experiment was conducted in lower pH buffer solutions (pH = 5.8 and 2.7) where large *T* values (> 90) for copolymer **3** is maintained at 40 ^oC. The increase in LCST compared to pNIPAAm is likely due to the incorporation of the more hydrophilic AEMA. Therefore, the temperature-dependent properties of pNIPAAm in copolymer **3** does not contribute at the temperature range used in our study. The above finding is in agreement with the work of Yavuz *et al*. who demonstrated that the incorporation of acrylamide (AAm) into pNIPAAm allows the LCST of pNIPAAm-*co*-pAAm to be tuned from 32 to 50 ^oC, depending on the amount of AAm.[1] When the amount of AAm is increased, the LCST is shifted to higher temperature. For example, for pNIPAAm-*co*-pAAm with a molar ratio of NIPAAm : AAm = 75% : 25%, the LCST is 48.9 ^oC.

Supplementary Note 2: We used the Higuchi model to describe the drug release kinetics.[2] This model, based on a diffusion controlled release of drug from a matrix system, is commonly utilized to describe the kinetics of drug release from mesoporous silica.[3,4] According to the Higuchi model, the amount of drug released after time *t* is

given by Q:

$$Q = \sqrt{\frac{D\varepsilon}{\tau}\left(2C - \varepsilon C_S\right)C_S t} \quad (1)$$

where D is the diffusion coefficient of the drug in the matrix solution, ε is the porosity of the matrix, τ is the capillary tortuosity factor, C is the total amount of drug in the matrix and C_s is the solubility of drug in the matrix solution. Therefore, for a diffusion controlled release of drug, Q displays a linear relationship with $t^{½}$.

Supplementary Fig. 4 shows a plot of Q *vs*. $t^{½}$ for the release of tobramycin based on Fig. 3B (main text). We note from Supplementary Fig. 4 that the antibiotic released from silica particles follows a two-step process; a fast release within the first 2 h followed by a slower release. For both steps, the Q *vs*. $t^{½}$ plot can be described by a linear relationship, suggesting a diffusion controlled kinetics for the release of tobramycin. The last point in Supplementary Fig. 4 at $t^{½}$ = 9.8 $h^{½}$ shows slight deviation from the linear fit since it is close to the time region when the amount of drug released has reached steady-state. The first (fast) step is likely due to the release of drug molecules that are either weakly bound to the silica pore surfaces or not efficiently encapsulated within the matrix,[5] whereas the second (slower) release step is due to drug molecules that interact relatively stronger with silica due to the amine groups on tobramycin. There is also a possibility of a small fraction the drug that is still tightly bound to the surface of the silica after a long time and remains trapped in the pores. Another plausible explanation of the two-step process could be due to different dissolution rates of silica.[3]

Supplementary Note 3: A tobramycin E-test assay was conducted to show that for *P. mirabilis,* the MIC value of tobramycin against the bacteria is unaffected by the presence of copolymer **3**. Using a tobramycin E-test strip, the MIC value of tobramycin against *P. mirabilis* on agar is determined to be ~ 2.0 μg mL^{-1} (Supplementary Figure 6A); in agreement with previously reported values.[6,7] In a separate experiment, *P. mirabilis* was applied onto an agar whose entire surface was first treated with 0.28 mg of copolymer **3** (*i.e.*, approximately twice the amount of

copolymer **3** attached onto the silica particles deposited on the left side of the agar in Fig. 4B of main text). In this case, the MIC value of tobramycin against *P. mirabilis* exposed to copolymer **3** is determined to be also ~ 2.0 μg mL^{-1} from the tobramycin E-test strip (Supplementary Figure 6B); indicating that the bacteria do not become more susceptible nor the activity of the antibiotic enhanced in the presence of the copolymer used here.

Supplementary Note 4: The control experiment in Supplementary Fig. 7 shows that when silica particles loaded with tobramycin but *without* a copolymer shell are deposited on the left half of the agar (4 %), immobile bacteria are able proliferate at the side of the agar that does not contain any particles but not at the side containing the drug-loaded particles. This is because tobramycin is released from the particles without an initial copolymer coat causing a sufficiently high concentration of free drug to be available at the left side of the agar to kill the bacteria.

Supplementary Note 5: Since the amount of peptide present on the agar in Fig. 4B (main text) is ~ 0.035 mg, the volume of water needed to reach a peptide concentration $\geq$ MIC of peptide against *P. mirabilis* (*i.e.*, 1.6 mg mL^{-1}) is at most 22 μL. The volume of water present on or near the agar surface is most likely to be higher than 22 μL which results in the actual concentration of the peptide on the agar to be smaller than its MIC value.

Supplementary Note 6: AFM force spectroscopy experiments were conducted to determine the adhesion forces between copolymer **3** (containing the peptide ligands) and SiO_2 (silica), lipopolysaccharide (LPS from *P. mirabilis*) and lipoteichoic acid (LTA from *B. subtilis*). Briefly, separate force curves were obtained for the interactions between bare SiO_2 spherical tip with copolymer **3**, LPS and LTA. It is observed that the interactions between bare SiO_2 tip and a layer of LPS and a layer of

LTA attached on the surface of a Si wafer are negligible (see Supplementary Fig. 9a and 9b for representative force curves).

In a separate experiment, a SiO_2 spherical tip functionalized with a shell of copolymer **3** (as per the mesoporous silica particles used to encapsulate drug) was used to determine the adhesion force between the copolymer and LPS/LTA. We note that there is no observable interaction between the modified tip and a layer of LTA attached on a Si wafer surface (see Supplementary Fig. 9c for a representative force curve), suggesting that the adhesion force between copolymer **3** and LTA is not measurable here.

In the case of LPS, for the 90 to 432 ramp cycles, the adhesion peaks for LPS become narrower and a much smaller force is seen (*e.g.*, 1.7 to 3.5 nN for the 3 representative curves in Supplementary Fig. 10A). After 432 ramp cycles, no adhesion peak is observed (Supplementary Fig. 10B). This is likely due to the peeling off of copolymer **3** from the contact surface of the SiO_2 tip as a result of the stronger adhesion force between the copolymer and LPS.

Supplementary Note 7: The MIC value of tobramycin against *B. subtilis*, as determined using the tobramycin E-test assay (Supplementary Figure 11A), is 1.5 μg mL^{-1} which is only slightly lower than the MIC against *P. mirabilis* (*i.e.*, 2.0 μg mL^{-1}). Therefore, a reasonable comparison of the effect of released tobramycin against the two bacteria can be made. In this case, an antimicrobial activity disk assay, similar to Fig. 4C of main text, was conducted on swarmer *B. subtilis* on agar (0.5 %) using 0.05 mg of copolymer **3**-coated silica particles containing tobramycin. It is worthwhile to note that differences in agar concentrations do not significantly affect the inhibition zone and antibiotic gradient of free tobramycin.[8] We observe that unlike for *P. mirabilis* (Fig. 4C), the swarmer *B. subtilis* colony expanded fully into the side of the agar containing the drug-loaded silica particles (Supplementary Fig. 11B). This result indicates that no lethal amount of drug is released from the copolymer **3**-coated silica particles despite the presence of motile *B. subtilis*.

Supplementary Figures

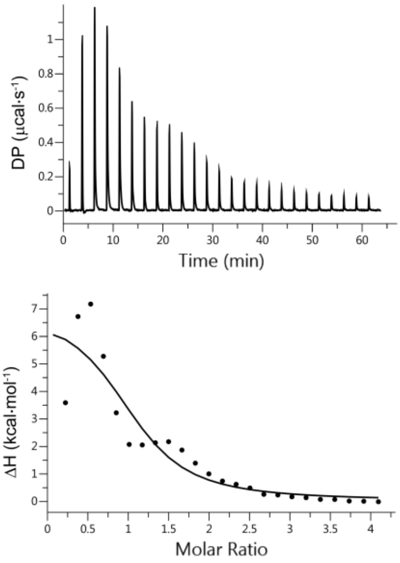

Supplementary Fig. 1 ITC thermogram of interactions between LPS (from *P. mirablis*) and the YVLWKRKRKFCFI-NH_2. The association constant K_a between YVLWKRKRKFCFI-NH_2 peptide and LPS was determined by fitting the ITC data into a single set of binding sites. A K_a value of 0.130 μM^{-1} is obtained.

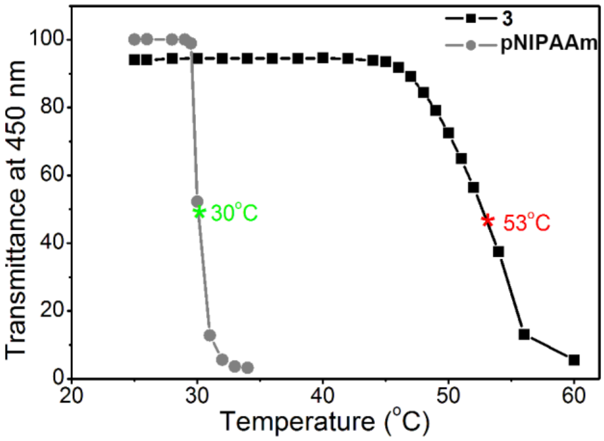

Supplementary Fig. 2 The transmission of pPNIPAAm (α,ω-bis(carboxy)-terminated) and copolymer **3** in PBS buffer (pH = 7.2) at 450 nm *vs*. temperature.

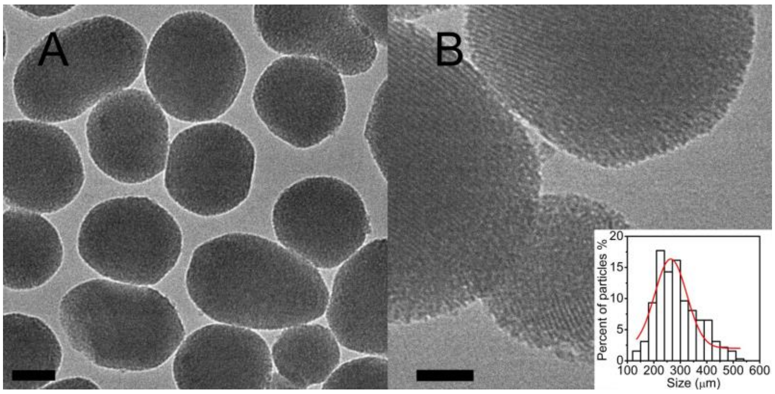

Supplementary Fig. 3 (A) The TEM image of MCM-41 silica particles. Scale bar: 100 nm. (B) The zoomed-in TEM image showing the mesoporous structure of MCM-41 silica particles. The pore size is 3.5 nm. Scale bar: 40 nm. Inset of (B) shows the histogram of the size (diameter) distribution of 322 particles. The mean diameter obtained from a Gaussian distribution fit is 264 ± 60 nm (s.d.).

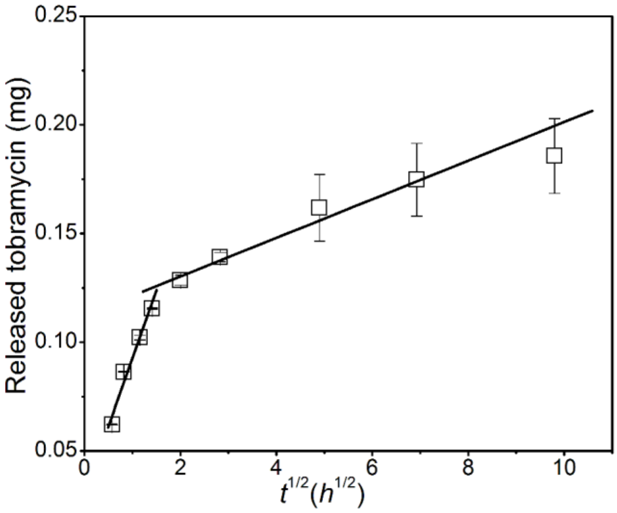

Supplementary Fig. 4 Amount of tobramycin released *vs.* square-root of time (based on Fig. 3B). The correlation coefficients of the linear fits for the first and second steps are R_c = 0.98 and 0.97, respectively

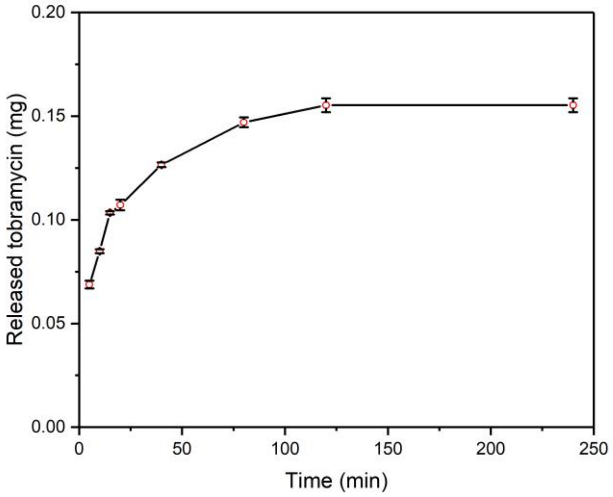

Supplementary Fig. 5 Drug release curve showing the amount of tobramycin released from drug-loaded mesoporous silica particles covered with a coat of copolymer **3** at various times. The drug is released when LPS-coated polystyrene latex beads are stirred with the silica particles at 100 rpm. The amount of drug released was determined using HPLC. Each data point is the average of two independent experiments and error bars represent SD.

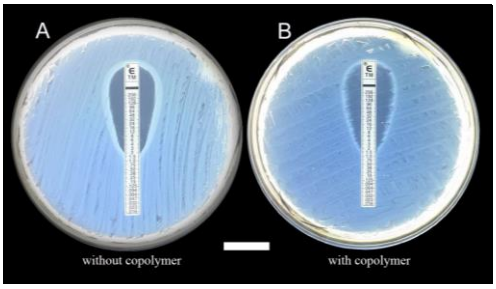

Supplementary Fig. 6 Tobramycin E-test of *P. mirabilis* in the absence (A) and presence (B) of copolymer **3**. Scale bar: 2 cm.

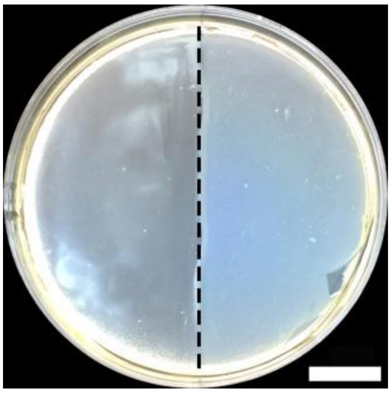

Supplementary Fig. 7 *P. mirabilis* cells were inoculated throughout the surface of a (4 %) agar and dried. Silica particles (0.2 mg) loaded with tobramycin but without a copolymer shell are deposited on the left half. The bacteria proliferated at the side of the agar that does not contain any particles (*i.e.*, opaque right half) but not at the side containing drug-loaded particles (i.e., significantly less opaque left half). In the absence of the copolymer shell, tobramycin is easily released from the silica particles into the agar. This experiment proves that the dosage of drug containing silica particles is large enough to inhibit proliferation of *P. mirabilis*. Scale bar: 2 cm.

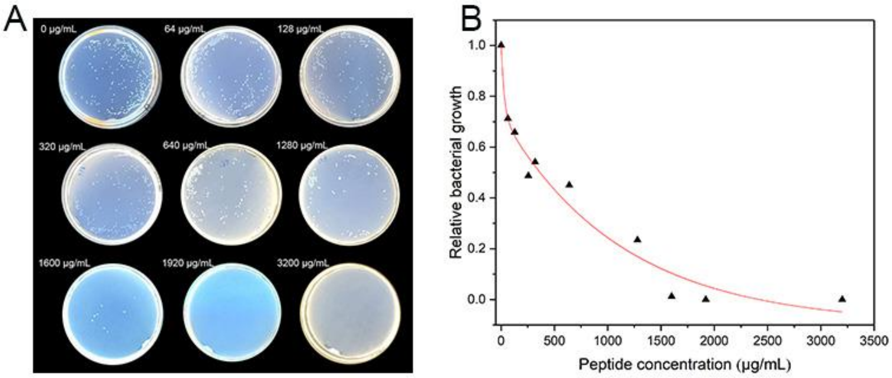

Supplementary Fig. 8 (A) *P. mirabilis* bacterial colonies on agar plate after treatment with various concentrations of peptide. (B) Relative bacterial growth after treatment with various concentrations of peptide with respect to growth in the absence of peptide. The curve was fit to a triple-exponential decay function and the minimum concentration of peptide needed to give 90 % growth inhibition is 1.6 mg mL^{-1}.

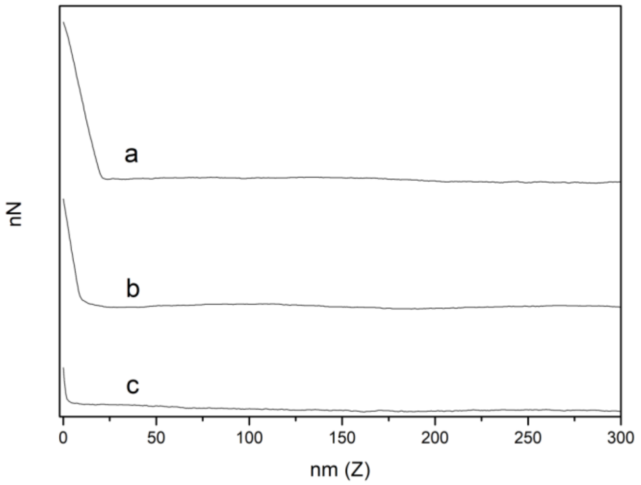

Supplementary Fig. 9 Representative force curves plotted for the interactions between a bare SiO_2 spherical tip and a layer of LPS (from *P. mirabilis*) (a) and LTA (from *B. subtilis*) (b) attached on a Si wafer. A representative force curve plot for the interaction between a copolymer **3** functionalized SiO_2 tip and a layer of LTA attached on a Si wafer (c).

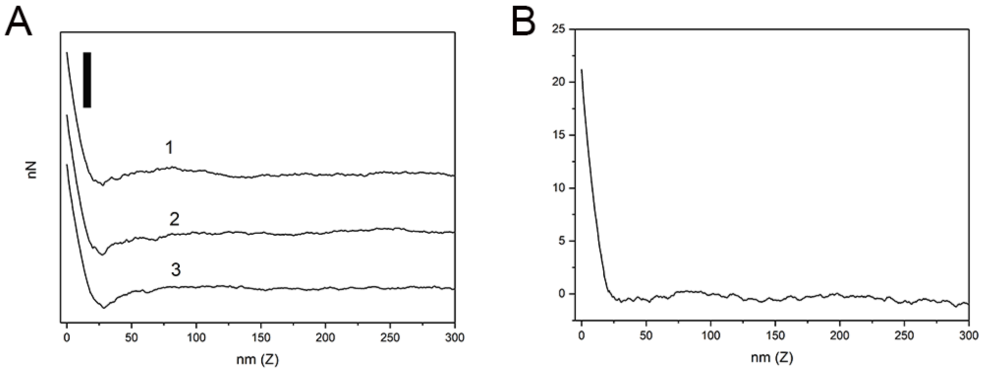

Supplementary Fig. 10 Representative force curves plotted for the interaction between a copolymer **3** functionalized SiO_2 tip and a layer of LPS (from *P. mirabilis*) attached on a Si wafer and collected 90 to 432 ramp cycles (adhesion forces for curves 1, 2 and 3 are 1.67, 3.60 and 3.18 nN, respectively) (A), and after 432 ramp cycles where no adhesion peak is seen (B). Scale bar: 10 nN.

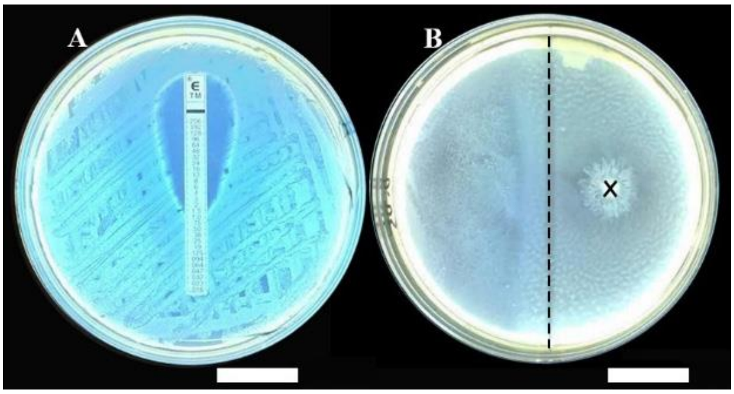

Supplementary Fig. 11 (A) Tobramycin E-test of *B. subtilis*. (B) Colony expansion of *B. subtilis* observed 20 h after cells were inoculated on 0.5 % agar at point x. 0.05 mg of copolymer **3**-coated silica particles containing tobramycin were deposited evenly on the left side of the agar surface. Scale bar: 2 cm.

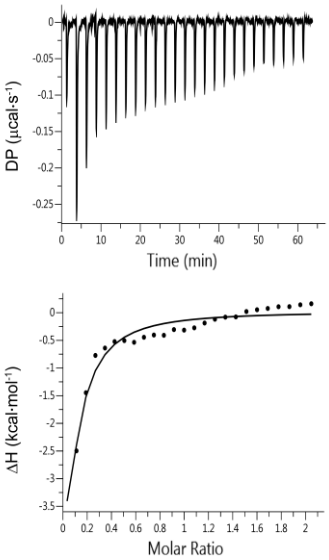

Supplementary Fig. 12 ITC thermogram of interactions between LTA (from *B. subtilis*) and the YVLWKRKRKFCFI-NH_2. From the ITC thermogram and fitting the ITC data into a single set of binding sites, a K_a value of 0.042 μM^{-1} is obtained.

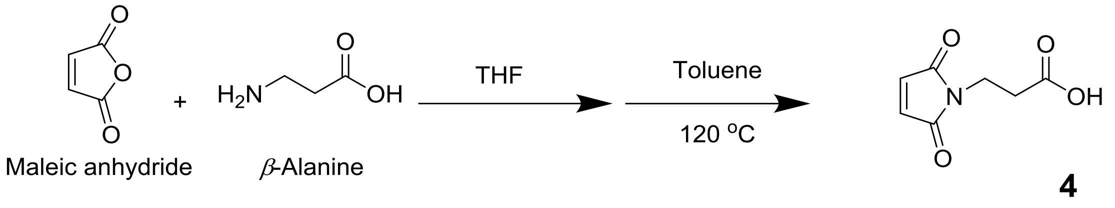

Supplementary Fig. 13 Synthesis of 3-maleimidopropionic acid **4**.

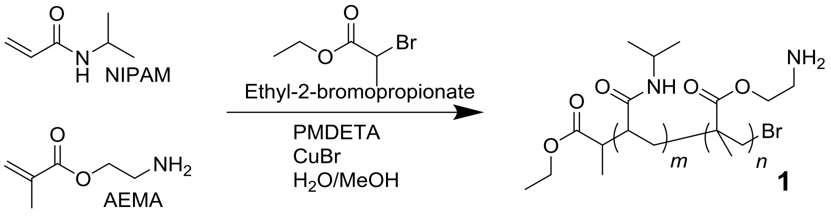

Supplementary Fig. 14 Synthesis of copolymer **1**.

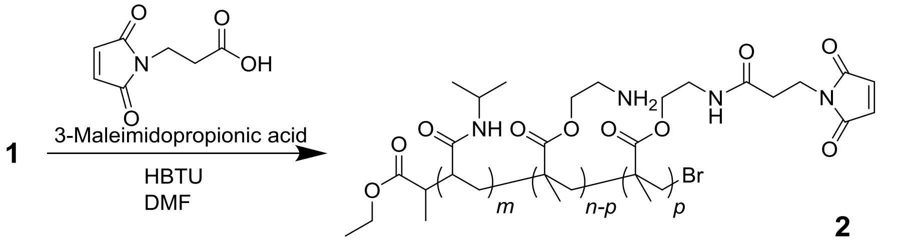

Supplementary Fig. 15 Synthesis of copolymer **2**.

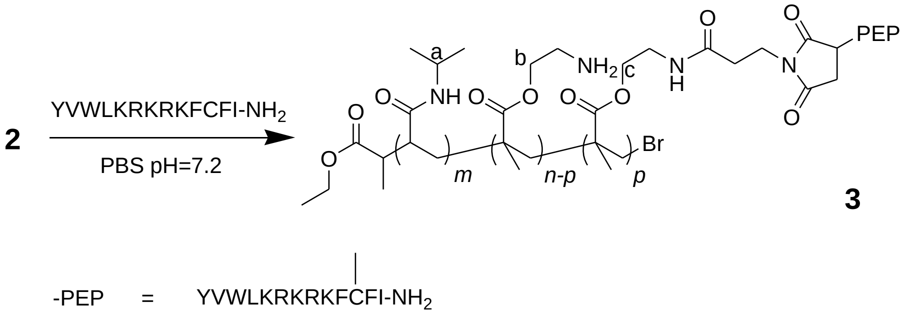

Supplementary Fig. 16 Synthesis of copolymer **3**.

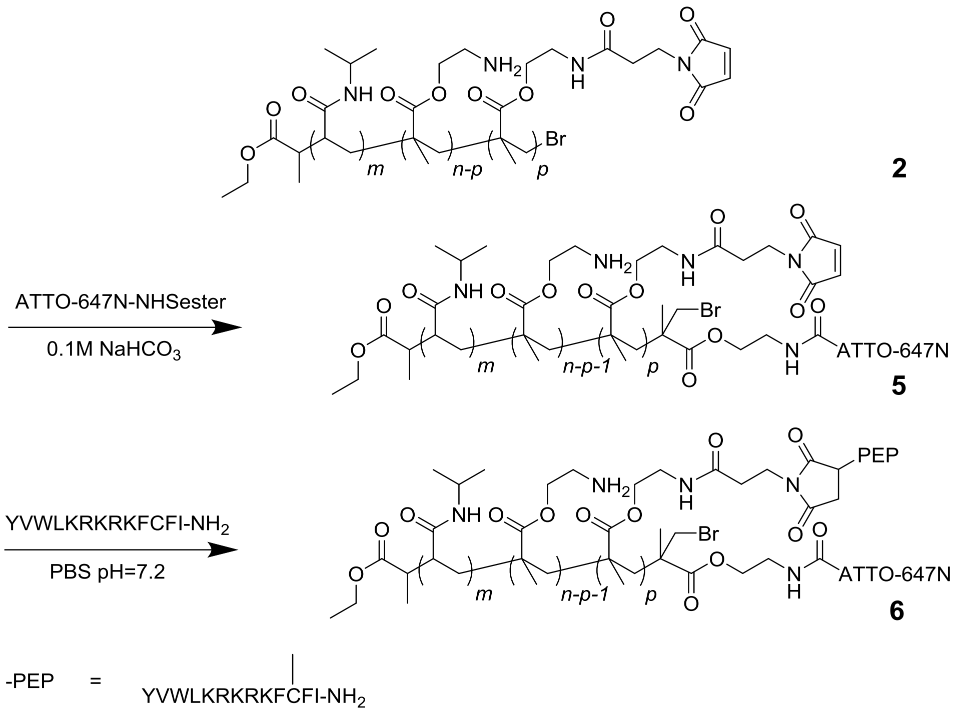

Supplementary Fig. 17 Synthetic steps to prepare copolymer **6**.

<u>Supplementary References</u>

1. Yavuz, M. S., Cheng, Y., Chen, J., Cobley, C. M., Zhang, Q., Rycenga, M., Xie, J., Kim, C., Song, K. H., Schwartz, A. G., Wang, L. V. & Xia, Y. Gold nanocages covered by smart polymers for controlled release with near-infrared light. *Nat. Maters*. **8**, 935-939 (2008).

2. Higuchi, T. Mechanism of sustained-action medication. Theoretical analysis of rate of release of solid drugs dispersed in solid matrices. *J. Pharm. Sci*. **52**, 1145-1149 (1963).

3. Andersson, J., Rosenholm, J. & Lindén, M. Mesoporous silica: an alternative diffusion controlled drug delivery system. In *Topics in Multifunctional Biomaterials and Devices Vol. I* (ed. Ashammakhi, N.) (World Scientific Publishing, Singapore, 2008).

4. Radin, S.; Chen, T. & Ducheyne, P. The controlled release of drugs from emulsified, sol gel processed silica microspheres. *Biomaterials* **30**, 850-858 (2009).

5. Kim, H. –W.; Knowles, J. C. & Kim, H. –E. Hydroxyapatite porous scaffold engineered with biological polymer hybrid coating for antibiotic Vancomycin release.

J. Mater. Sci. Mater. Med. **16**, 189-195 (2005).

6. Takahashi, Y., Okabe, T., Takeuchi, H., Daijoh, K. & Ohshiro, K. Clinical evaluation of tobramycin. *Chemotherapy* **23**, 1290-1296 (1975).

7. Britt, M. R., Gabribaldi, R. A., Wilfert, J. N. & Smith, C. B. In vitro activity of tobramycin and gentamicin. *Antimicrob. Agents Chemother*. **2**, 236-241 (1972).

8. Lai, S., Tremblay, J. & Déziel, E. Swarming motility: a multicellular behavior conferring antimicrobial resistance. *Environ. Microbiol*. **11**, 126-136 (2009).
