# Supplementary material for: Lipopolysaccharide-affinity copolymer senses the rapid motility of swarmer bacteria to trigger antimicrobial drug release
Source: Nat Commun. 2018 Oct 15;9:4277. doi: 10.1038/s41467-018-06729-6 (PMC6189052; doi:10.1038/s41467-018-06729-6)
Supplement: Supplementary file 3 — Description of Additional Supplementary Files [file 41467_2018_6729_MOESM3_ESM.docx]

Description of Additional Supplementary Files

**Supplementary Movie 1.** Continuous wide-field fluorescence images of swarmer P. mirabilis cultured on an (1 %) agar in the presence of mesoporous silica particles with dye-labelled copolymer 3 attached on the surface. The particles are not loaded with drug. In this video, white light is used to image the bacterial colony and red light (633 nm light) is used to image dye-labeled copolymer 3. The illumination source was alternated between white light and red light with white light illumination turned on at 0.588, 4.475, 9.604 and 15.909 s, and red light at 2.646, 7.416 and 11.989 s. During red light illumination, bright swirling streaks are observed which are due to detached copolymer 3 moving within the swarmer colony. During the transition from one light source to the other, a superposition of the images of the colony (from while light) and dye-labeled copolymer 3 (from red light) can be observed. The dimension of the image is 120 × 120 μm^2^.

**Supplementary Movie 2.** Continuous wide-field fluorescence images of multi-layered swarmer B. subtilis cultured on an (0.5 %) agar in the presence of mesoporous silica particles with dye-labelled copolymer 3 attached on the surface. The particles are not loaded with drug. In this video, white light is used to image the bacterial colony and red light (633 nm light) is used to image dye-labeled copolymer 3. The illumination source was alternated between white light and red light with red light illumination turned on at 0.561, 11.473, 20.868 and 31.615 s, and white light at 9.791, 15.527 and 25.220 s. During red light illumination, no obvious bright streaks corresponding to copolymer 3 chains being carried away from the particles are seen. The dimension of the image is 120 × 120 μm^2^.

**Supplementary Movie 3.** Continuous wide-field fluorescence images of single-layered swarmer B. subtilis cultured on an (0.5 %) agar in the presence of mesoporous silica particles with dye-labelled copolymer 6 attached on the surface. The particles are not loaded with drug. In this video, white light is used as the illumination source for the first 3 s, and a typical single-layered swarmer colony is seen. At 3 s, the illumination source is switched to a 633 nm light that excites Atto 647N molecules. No obvious bright streaks corresponding to copolymer chains being carried away from the particles are seen. The dimension of the image is 120 × 120 μm^2^.
